# Between Protest and Counter-Expertise: User Knowledge, Activism, and the Making of Urban Cycling Networks in the Netherlands Since the 1970s

**DOI:** 10.1007/s00048-022-00341-y

**Published:** 2022-08-09

**Authors:** Henk-Jan Dekker

**Affiliations:** Eindhoven University of Technology, Eindhoven, The Netherlands

**Keywords:** urban cycling, activism, counter-expertise, user knowledge, Netherlands, städtisches Radfahren, Aktivismus, Gegenexpertise, Benutzerwissen, Niederlande

## Abstract

Around 1970, high numbers of traffic casualties among cyclists led to the creation of numerous local protest movements in the Netherlands. While activists employed protest strategies, their main interest lie in the way they exemplify a highly successful instance of “lay expertise”; the idea that users of a technology have a fundamentally different and valuable perspective on a technology than experts or system-builders. Specifically, cyclists claimed to be more knowledgeable about cycling conditions and safety than the state-employed engineers and traffic experts who built the roads and cycling path network. A key actor in this story is the Dutch Cyclists’ Union (*Fietsersbond*), a national platform of local action groups formed in 1975. These activists used the cycling experience of everyday utilitarian cyclists to compile maps and blacklists of locations where cycling was dangerous, unpleasant, uncomfortable, or otherwise discouraging. In doing so, they successfully claimed legitimacy as a valuable knowledge partner for local engineers and policymakers. As a result, they gained some level of influence within local governments, a relation which in the intervening years has only grown stronger. This case study shows how users can shape socio-technical systems bottom-up, and can therefore to an extent be seen as a successful example of co-construction of technology.

## Abstract (de)

Um 1970 führte die hohe Zahl von Verkehrsunfällen unter Radfahrern zur Gründung zahlreicher lokaler Protestbewegungen in den Niederlanden. Obwohl die Aktivisten auch Proteststrategien einsetzten, besteht das Hauptinteresse dieses Artikels in der Art und Weise, wie sie ein äußerst erfolgreiches Beispiel für „Laienkompetenz“ darstellen. Die Idee dahinter ist, dass Nutzer (einer Technologie) eine wertvolle und grundlegend andere Perspektive auf eine Technologie haben als Experten oder Systembauer: Radfahrer gaben in Umfragen an, mehr über die Bedingungen und die Sicherheit von Fahrrädern zu wissen als die staatlich angestellten Ingenieure und Verkehrsexperten, die die Systeme des Straßen- und Radwegenetzes gebaut haben. Ein Hauptakteur dieser Geschichte ist der *Fietsersbond* [Radfahrerbund], eine nationale Plattform lokaler Aktionsgruppen, die 1975 gegründet wurde. Diese Aktivisten nutzten die Mobilitätserfahrung täglicher Radfahrer, um Karten und Liste von Orten zusammenzustellen, an denen das Radfahren gefährlich und unangenehm oder auf andere Weise beschwerlich war. Damit beanspruchten sie erfolgreich Legitimität als wertvoller Wissenspartner für lokale Ingenieure und politische Entscheidungsträger. Infolgedessen gewannen sie einen gewissen Einfluss auf die Kommunalverwaltung; eine Beziehung, die seitdem immer stärker geworden ist. Diese Fallstudie zeigt, wie Nutzer sozio-technische Systeme „von unten“ gestalten können; sie kann daher in gewissem Maße als erfolgreiches Beispiel für die kooperative Konstruktion von Technik angesehen werden.

Across the western world, the 1970s were marked by an emerging environmental consciousness and critiques of capitalism. The role of the automobile in the transformation of cities into polluted, dangerous, and unlivable areas formed one particularly contentious issue. Cycling activists in the Netherlands were connected to a transnational activist culture taking aim at automobility and environmental degradation in a context of anti-capitalist critique. The works of urban theorist Jane Jacobs (*Death and Life of Great American Cities, *1961), economist Ernst Schumacher (*Small is Beautiful, *1973) and social critic Ivan Illich (*Energy and Equity, *1974) inspired these activists (Furness 2010: 66). In some countries (e.g., the US) activists preferred to organize public demonstrations and media spectacles, which proved more efficient than working with local government (Furness 2010: 64). In a number of European countries, however, political traditions were more consensual and corporatist, meaning that state officials saw certain interest or lobby groups as representatives of major societal interests. Believing it important to gain support for policy by negotiating with these societal groups, certain interest groups became important intermediaries between citizens or users and the government. Scholars have observed this type of relationship between cycling activists and government in a number of European countries like Denmark, Switzerland, Sweden and the Netherlands (Balkmar 2020: 337; Micheletti 1991; Emanuel 2019; Mijnssen-Hemmi & Boller 2006). This article places Dutch cycling activism in this context, and analyzes its key action strategies and successes. It thus forms a contribution to the emerging, but still scant scholarship on anti-car and pro-cycling activism in Europe and the US in the 1970s and beyond.

While the Netherlands broadly shares certain political characteristics with other small European democracies, some of its mobility conditions are more unique. While over the course of the 1960s, cycling had declined in many European countries, in the Netherlands it still held a significant modal share. Whereas outside cities, provision of separate cycling infrastructure had a long history, urban infrastructure had not been provided (Albert de la Bruhèze et al. 1999; Ebert 2010; Oldenziel et al. 2016; Dekker 2021). When Dutch car ownership increased rapidly in the 1960s (later than elsewhere) the large group of existing cyclists clashed with drivers. The safety of many cyclists, not least school children, came under threat. This threat provided an even more pressing issue than elsewhere, where cycling activism had been linked to somewhat less visible environmental issues like pollution, as Simon Gunn argues for the UK, and Zack Furness for the US (Gunn 2018; Furness 2010). This is not to say that ecological arguments were absent from the Dutch activist scene; cycling advocates often referred to cyclists’ efficient use of energy and space. However, the safety argument made Dutch cycling activism simultaneously more urgent and political, since human lives were directly at stake, but, in another sense also less political, since it was an issue that transcended political divisions.

The divergent national trajectories of cycling—and the various development paths of automobility—in the twentieth century meant different national cycling cultures were formed. As Albert de la Bruhèze, Veraart and Ebert have argued, in the Netherlands non-governmental organizations had historically worked closely with the Dutch state to achieve a system in which (suburban and rural) cycling and automobility could co-exist. Starting in the 1920s, the Dutch tourist organization ANWB (*Algemene Nederlandse Wielrijdersbond*, 1883–now) lobbied for separate cycling paths alongside major roads as well as a network of recreational cycling infrastructure in the countryside. In a political compromise, engineers used the Dutch bicycle tax (levied from 1926 to 1941) to (re)construct car roads but felt obliged to provide these with cycling paths in exchange for cyclists’ tax money (Albert de la Bruhèze & Oldenziel 2015; Ebert 2010; Ebert 2012).^1^ While bicycle taxation existed in other European countries, only in the Netherlands was it then spent directly on cycling infrastructure. This partly explains the longevity and durability of Dutch cycling culture. Engineers implemented this policy by expanding access gradually in the 1950s and 1960s. They built on the interwar efforts of the the tourist organization ANWB and private organizations to build recreational cycling paths. In the post-war Netherlands, state actors (provinces) took over this role. As a result, by 1970, there were many cycling facilities, but crucially, urban planners and engineers had done very little to protect urban cyclists against the ever-growing space demands of the car.

The urban conflict over space came to a head in the second half of the 1960s as car levels suddenly rose sharply alongside steady cycling levels. While the Netherlands eventually reached similar levels of automobility as other European countries, this happened comparatively late. In 1960, the Netherlands had 45 cars per 1,000 inhabitants, lower than Belgium (82) or Switzerland (89) while the number in France and Great-Britain was over a 100, and in the United States it was almost 350. Many more people therefore did not own a car, but their needs did not receive nearly as much attention from policymakers and engineers (Gunn 2013). By 1970 Dutch car ownership reached similar levels to those of Belgium and Switzerland at circa 200 cars per 1000 inhabitants (Filarski & Mom 2011; Wolf 2010). This late development is partly explained by the end of the Dutch policy of controlled salaries in the early 1960s, which created a sudden increase in purchasing power resulting in a rapid increase in car ownership. Another factor was the continued use Dutch people made of the bicycle: at the lowest point in Dutch urban cycling (around 1970),the bicycle still had a modal share of roughly 30% in many cities (Albert de la Bruhèze et al. 1999; Oldenziel et al. 2016: 13; Stoffers 2021). Where traffic was not separated, these two modes were on a collision course. By 1970 more than 500 cyclists and more than 400 children under the age of 14 were killed in traffic every year.^2^ As a result, Dutch urban cyclists took to the streets in an attempt to convince the government to address this social issue. From the start, however, they complemented their protest strategies with strategies of counter-expertise. This latter strategy quickly became dominant.

In consensual democracies like the Netherlands, there is a precedent for consulting non-governmental stakeholders in policymaking. This encourages activist groups to take a pragmatic and expertise-driven approach to advocacy work, in which the production of (lay) expertise and cooperation with the government are key. The transformation of Dutch cities from car-dominated into more bicycle-friendly environments is an important example of how lay expertise and bottom-up activism can shape (traffic) environments. This stands in stark contrast to Anglo-Saxon countries with low levels of cycling, where user expertise is often not available to the same extent, and the state is unwilling to take cycling action groups seriously. As a result, protest seems a much more apt strategy (Furness 2010: 64; Longhurst 2015; Mohl 2008). American cycling activists of the 1970s recall their experiences of being firmly outside the system or even marginalized (Furness 2005: 67). Nor are companies central to Dutch activism as they were in France. In his study on the role of French NGOs in cycling governance since the 1970s, Maxime Huré shows how (transnational) networks of interest groups and enterprises sought out public-private partnerships to improve cycling at the local level in France (Huré 2013). As I show, activism in the Netherlands was bottom-up and broadly supported, whereas the non-governmental actors in France were primarily advertising companies and joined by a smaller cadre of lobbyists.

Although social unrest over car-centric cities was widespread in the 1970s, research into the topic is still in its infancy. As Harald Engler notes in a recent article on German protest movements, “resistance towards the car-friendly city …is still under-researched.” (Engler 2020: 354) Even within the historiography of social movements in the Netherlands, surprisingly few researchers have studied activism around cycling and traffic safety. A 1992 book claiming to give an overview of the history of Dutch social movements does not include any of the many action groups around cycling and mobility in general (Duyvendak et al. 1992; Kriesi 1989). The historiography of the Dutch environmental movement also rarely mentions cycling activism (Cramer 1989; Van der Heijden 1992). An exception is a 1978 collection on environmental activism in the Netherlands containing two pieces by (part-time) activists (Udo de Haes & ter Keurs 1978; Kalk 1978) as well as the work of Manuel Stoffers on the human-powered vehicle movement (Stoffers 2016; Stoffers 2019; Stoffers 2021). The only work on the Dutch Cyclists’ Union is a report commissioned for its thirtieth anniversary in 2005, written by Bram Duizer (Duizer 2005). Duizer observes a pragmatic turn soon after the constitution of the Cyclists’ Union, a point I substantiate and elaborate on in this paper. As I will demonstrate, the relative dearth of publications on Dutch cycling activism is a major oversight, since the Cyclists’ Union was formed through the cooperation of a large number of local mobility-related protest groups. This activism was widespread in Dutch society and deserves more attention.

While the social movements scholarship neglects cycling activism, historians of cycling have acknowledged the important role of social movements. In *Cycling Cities*, Ruth Oldenziel, Martin Emanuel, Adri Albert de la Bruhèze and Frank Veraart and their co-authors direct their attention to the role of activists. Based on the pioneering work of Veraart and Albert de la Bruhèze (1999), the authors describe a wave of protest movements that began in the late 1960s and gained momentum in the 1970s, only to be frustrated by the funding cuts of right-wing governments in the 1980s (Oldenziel et al. 2016). Whereas *Cycling Cities* predominantly focuses on the protests of the action groups, I argue that expertise-based activism was in fact the dominant action strategy of the Cyclists’ Union.

In contrast to this scarcity of scholarship on Dutch cycling activism, the role of lay people or lay experts in contesting and (co)producing scientific expertise has been well researched within Science and Technology Studies (e.g., Nelkin 1975; Wynne 1989; Wynne 1996; Epstein 1995; Callon 1999; Turner 2001; Grundmann 2017; Kuchinskaya 2019). Activism in health domains (such as around the AIDS crisis) or energy (around nuclear power plants) are often the case studies of choice. The activism in those cases involved the use of counter-experts to challenge the dominant discourse. While the core of Dutch cycling activists was often academically trained, they organized the production of counter-expertise by collecting the experiences of everyday cyclists. In this way, they form an interesting example of bottom-up production of knowledge. Dutch planners, who had been preoccupied with facilitating car use, had neglected to gather this type of information and were consequently not in a position to adequately design safer cycling facilities. In the political climate of the early 1970s opinions on the design of urban space and the place of the car in it changed, leading these planners to begin incorporating the data gathered by activists. My argument is therefore that the production of counter-expertise played an important role in attempts at making Dutch cities more bicycle-friendly that have been ongoing since the 1970s. In what follows, the first section shows how countercultural movements helped put cycling on the political agenda, while contrasting their action strategies and relation to the government to that of later activists. The second section explores how the action group *Dooievaar* pioneered the strategy of cycling counter-expertise, while the third section shows how this methodology was applied by the Dutch cycling activist group Cyclists’ Union, founded in 1975. The final section concludes with some thoughts on why this type of activism was successful in the Dutch context.

## Protest Strategies

While cycling was only a minor part of their activities, the early countercultural Provo and Kabouter (Gnome) movements paved the way for later cycling activists by formulating a trenchant critique of car culture (on Provo see Van Duyn 1985; Mamadouh 1992; Kennedy 1995; Pas 2015; Otten 2017; on Kabouter: Tasman 1996). The subversive anti-capitalist youth movements Provo (1965–1967) and Kabouter (1969–1974) criticized capitalism’s materialist consumer culture, of which the car was a powerful symbol. They charged that the city had become a place of (large-scale) consumerism rather than a living environment with a mixture of functions, as Jane Jacobs had already argued. The car, with its large space demands and polluting exhaust fumes, became the objects of these activists’ ire. In contrast to later activists, their actions were typically provocative and playful and contributed more to a mentality change than concrete transformations of urban space.

Provo activists—inspired by French Situationist thinkers of the 1950s—were among the first to draw attention to the problem of traffic problems and safety in the Netherlands (Furness 2005: 402). The White Bicycle Plan (1965), a plan to introduce free-to-use shared bicycles in the city, is well known as an early and effective critique of the polluting and space-consuming role of the car in the city (Van Duyn 1985: 75–76; Mamadouh 1992: 67–68; Furness 2010: 55–58; Feddes & De Lange 2019: 58–62). Besides politicizing the bicycle, it also marked the invention of the shared bicycle (Ploeger & Oldenziel 2020). One Provo member Thom Jaspers—described in the press as the “safe traffic magician” (*veiligverkeermagiër*)—organized happenings around traffic safety in 1965. Shortly before, Jaspers witnessed an accident which made him see traffic as “a pagan god” to which “we Dutch people sacrifice seven people every day.”^3^ The first happening took place at a small art gallery in Schiedam near Rotterdam and it involved Jaspers’ Japanese friend the performance artist Yoshio Nakajima pouring water and red paint over himself to symbolize the tears and blood of traffic victims while standing on a car wreck. Jaspers and others then demolished the car with metal poles.^4^ This seems to have been a relatively isolated action. While some of Jaspers’ rhetoric is reminiscent of the language the traffic safety action group Stop de Kindermoord (Stop the Child Murder) employed, it is hard to determine whether there is a direct connection. What is clear, however, is that in the early 1970s the anger over traffic accidents reached a critical mass and formed a broad societal basis for an action group that had not yet appeared on the scene.

After the demise of Provo, in 1969/1970 a similar movement emerged as the Kabouter (Gnome) movement. This group was ambitious in its vision for social transformation. They espoused a vision of transportation centered around free public transit. They also supported cycling, e.g., with their *Kabouterfietsclub* which promoted the cargo bike as alternative to the car (Tasman 1996: 61, 154–58; Mamadouh 1992: 105). In early 1970, the Kabouters organized multiple protest rides with regular and cargo bikes (Tasman 1996: 155). Apart from the excessive use of space, air pollution was a key complaint; in one demonstration, Kabouters pretended to faint and suffer oxygen deprivation because of car exhaust fumes.^5^ The *Auto-Eliminatiedienst *(Car Elimination Service) organized protests against the dominant place of the car in the city. They blocked traffic in streets like the Leidsestraat in Amsterdam where exhaust fumes and parked cars were a major problem with sit-ins. Despite confrontations with angry drivers and police arrests, these protests ultimately convinced the city to experiment with closing this street to car traffic (Tasman 1996: 157–58). Even these rather polarizing activists succeeded in wresting some concessions from the urban government.

The contribution of the countercultural movement to cycling in the long run consists primarily in a reframing of urban streets as public spaces rather than monofunctional spaces for cars. Movements like Provo and Kabouter that emerged across Europe sought to make the bicycle into what historian Zach Furness has called “a technological embodiment of environmentalism” (Furness 2010: 59). Many environmental protest groups did not explicitly strive for better cycling conditions, yet they nevertheless used the bicycle in demonstration rides (Levels 2020: 389). The car became a key symbol of everything wrong with capitalism while the bicycle presented a logical alternative which became the symbol of a different economic and ecological system. Protesters like those initiated by the Kabouter movement in Amsterdam popularized the idea of making certain streets car-free and giving cycling and public transit a larger role in urban mobility. New actions group that formed in the seventies shared the key values that Provo and Kabouter represented, emphasizing self-governance, livability, the small (neighborhood) scale, and walking and cycling and opposing to bureaucracy, unrestricted economic growth and the redevelopment of inner cities to accommodate big business and cars.^6^ Among urban social movements, cycling emerged as an antidote to many of the problems plaguing the city.

The relationship of early groups with the government was more strained than that of later action groups. Beginning in the mid-1970s, many cycling activists found it useful to work with the government. Within the Provo and Kabouter movement, however, entering into government institutions and dialogue with civil servants and politicians was highly controversial. Most preferred confrontational, non-parliamentary activism. When it nevertheless decided to participate in the 1970 municipal elections, the Kabouter movement won four seats in Amsterdam; in other Dutch cities they also had electoral successes. Roel van Duyn, the Kabouter’s leader in the Amsterdam city council, submitted numerous policy proposals. Ultimately, the strategy of deploying counter-expertise and working with the government became dominant in the Netherlands. This method achieved results: policymakers and engineers were open to the ideas of activists and shared the conviction that the car had taken over urban space to an unacceptable extent.

## Pioneering Counter-expertise Approaches

In the early 1970s, new action groups embraced a different approach centered around citizen participation and counter-expertise. An action group from The Hague pioneered an approach focused on collecting the experiences of normal everyday cyclists and demanding action on very concrete issues that was later adopted by the Cyclists’ Union. A group of four young architecture students concerned with city planning (Hans van Beek, Leo Hamer, Jan Ledderhof and Arij van der Stelt) founded “Dooievaar”*—*a contraction of the Dutch words for “dead” and “stork” the stork being the symbol of the city of The Hague—in 1972.^7^ Around this time, young architects around the world started to promote a new mode of urban planning, influenced by the work of Jane Jacobs and Jan Gehl (cf. Klühspies 2015). Their action repertoire, as Feddes & De Lange (2019) have also clearly demonstrated for Amsterdam, was very influential within Dutch cycling activism.

The architecture students worked closely with Eisse Kalk, an advocate for democratization in politics within the action group Werkgroep 2000 which aimed to increase citizen participation in what it considered to be technocratic planning traditions. The thesis project of the four students—which dealt with the social responsibility of architects and planners—was inspired by Kalk and the group corresponded with him about it. In Kalk’s view, local officials prepared plans in a non-transparent way and only allowed participation when most fundamental decisions had already been made and only the details remained to be tweaked.^8^ He argued that citizens should not only be allowed to comment on already developed plans, but should also be included earlier in the process, when an open discussion about the underlying assumptions and objectives of the plans should be held. These implicit aspects of the planning process, which were often couched in technical terms, hid fundamental political choices and trade-offs regarding urban planning and mobility from public view. In short, Kalk wanted to politicize local affairs, to make the trade-offs and priorities visible for everybody, and to incorporate the knowledge citizens had about their everyday living environment in plans to improve that environment.^9^ The design of public space and traffic policy was an area particularly suited to democratic procedures. Writing in 1978, Kalk noted that “there is no other terrain where action groups have shot up like mushrooms in the last 10 years outside traffic, transport and spatial planning.” (Kalk 1978: 62). According to him, the groups’ members came from all ideological backgrounds, precisely because problems with traffic and the living environment were so visible in everyday life.

Kalk’s ideas strongly influenced Dooievaar activists who used them to make two central claims, one about the process of policymaking, and one about its contents. Regarding the process, Dooievaar was a staunch defender of participation versus what they considered to be the technocratic planning of The Hague. Their central demand was an end to “authoritarian plan making.”^10^ In their thesis, the four students emphasized the political responsibility of architects and planners. Instead of blindly executing the planning and mobility vision of a small group of policymakers, architects and urban planners should be more politically and socially engaged. The goal of the transformation of the ethos of engineering was “a process of planning preparation in which the influence of the user/inhabitant is as large as possible.”^11^ The current Dutch transport planning was, in short, “one-sidedly technical.”^12^

Secondly, content-wise, the activists claimed that car-centered planning made cities unlivable and cycling an unattractive option. Dooievaar feared the transformation of The Hague into an American-style car city.^13^ In their thesis of 1972, the four students criticized an “Exploratory Memorandum for Slow Traffic” by the city planning department for containing too little in the way of concrete measures for cyclists, as well as for being narrowly inspired by traffic safety concerns which “in fact emphasize the subordinated position of cycling.”^14^ Even if the proposed measures might make cycling safer—something they questioned—they would also make it less attractive, when it was in the interest of quality of life in cities to promote cycling.^15^ Dooievaar criticized the city planners of The Hague for presenting better cycling routes as technically impossible and obfuscating the fact that the choice in favor of the car was an ideological one.^16^ The prioritization of the car was visible everywhere in the city and meant that the car always had the shortest route, while cyclists were presented with many barriers, detours, and long waiting times, not to mention the danger of cycling in a car city.^17^ This critique of official planners inability to plan a livable city for people echoes Jane Jacobs’ critique of American city planning (Furness 2010: 53; Kaal 2011: 537).

To draw attention to these issues, Dooievaar activists knew they had to grab the attention of politics and media, for instance through protests. In June 1973, 1,500 protesters attended a Dooievaar-organized demonstration.^18^ Among the attendees were member of parliament and former Minister of Public Works Willem Drees Jr., as well as The Hague alderman W.H.A. Nuy and multiple council members.^19^ Media strategies were important to Dooievaar and other activists, and good contacts with journalists helped them receive extensive coverage to publicize their ideas.^20^ The group also organized exhibitions and other events to inform the general population of the harmful effects of government plans. In one case they worked together with a group of biologists of Leiden University to successfully block the construction of a major road between Leiden and the Hague. Here, they practiced their ideal of democratization by informing road workers who were already contracted to build the road that some of their own homes would have to be demolished for the building project.^21^

Having secured an audience, Dooievaar continued to promote their message that citizens’ lay expertise should form the basis of plan-making. To show that this was possible, the activists devised a step-by-step plan for other activists published in the form of a manual.^22^ In this plan, they demonstrated how an ideal cycling route network could be drawn up with the input of everyday cyclists. In 1974 Dooievaar applied this to The Hague, and then published it in *Katernen 2000*, the magazine of the democratization activist group Werkgroep 2000.^23^ The steps other activists were advised to follow in their respective cities were, first, to identify the main areas of work, recreation, and living on the map and secondly, to deduce the main cycling routes necessary between them. The third step was to map these main routes onto actual streets, thus creating a cycling route network on paper. After this, the plan had to be published in local newspapers in order to elicit feedback from cyclists. After receiving user input, the fifth step was the creation of a final plan, including technical drawings of the ideal cycling path. Finally, this report then had to be used to enter a dialogue with local policymakers. Dooievaar included a detailed and knowledgeable breakdown of the inner workings of local city bureaucracy and politics. In this way, other activists received valuable pointers on how to engage the local citizenry and politicians into a conversation about better cycling facilities. The final result—a cooperation between educated traffic planners like the activists of Dooievaar and ordinary cyclists—was meant to be both an inclusive, as well as expertly-designed cycling route plan.^24^

In a book on Amsterdam’s cycling history, writer Fred Feddes characterizes Dooievaar’s activism as “depoliticization,” an expertise-based approach suggesting concrete improvements but eschewing open confrontation or a fundamental system critique (Feddes & De Lange 2019: 73). While my analysis of Dooievaar mostly confirms their interpretation, I come to a somewhat different conclusion. The emphasis on democratization and increased participation in the planning procedure in itself was a political confrontation with an older way of doing politics. In many documents the activists did plead for a political choice for the bicycle instead of the car. What is more, Dooievaar’s central point of contention involved the depoliticization of local planning, wherein planners followed “facts” without ever considering their desirability and the possibility of other policy goals. Dooievaar was interested in pragmatic solutions and believed it found it in a sort of co-design procedure for city planning. This was ultimately a procedure aimed at achieving consensus through deliberation that involved the entire city population in an explicit discussion of policy goals and trade-offs. As such, it formed an influential blueprint for Cyclists’ Union activism.

## Cyclists’ Union Activism: Goals and Methods

A number of action groups, consisting of both local cycling activists and national environmental action groups, came together in 1975 to create the Cyclists’ Union as a national platform.^25^ This national platform stimulated the exchange of best practices and tried to coordinate actions, but the dominant organizational model was that of local branches advocating for improved cycling conditions in a specific city or region. The most active members of the Cyclists’ Union tended to be young (in their twenties or thirties), university-educated, and with left-leaning political sympathies. For instance, the founding members of Dooievaar were young architecture students: Eisse Kalk had studied human geography and political science, Marten Bierman of *De Lastige Amsterdammer *was an architecture student, another key figure in that action group was the sociologist Henk Bakker. Many of the members engaged with new ideas about politics and city planning.

This is in line with what political scientist Hanspeter Kriesi has concluded about New Social Movements in the 1980s in general: their support was broad, but the active core was constituted by more radical and progressive members of the middle class (Kriesi 1989: 1102). Support for action groups like the Cyclists’ Union or Stop de Kindermoord was large, and while the core of the active membership might have belonged to a specific demographic, the support for traffic safety and cycling was much more widespread. As an illustration, the 55-year-old businessman F.H. Markerink volunteered in 1975 to organize a Cyclists’ Union branch around Enschede. His only daughter had been killed in a car accident and, although he did not want the Cyclists’ Union to become “too political,” he also thought the existing Cycling Safety interest group VVN (*Veilig Verkeer Nederland*) to be too cautious in its lobbying activity for traffic safety.^26^ For action groups to be successful, it is important that they can credibly claim to be the true representative of the people whose interests they defend. In the case of the Cyclists’ Union, the many groups that constituted it, the founding of numerous local branches, as well as the decade of cycling-related activism made this claim plausible to a broad public.

The main claim of the Cyclists’ Union was that the Dutch government was negligent in protecting “weaker” participants in traffic and “captive” cyclists against the hostile environment of car-centric cities. In a programmatic text from 1978, the Cyclists’ Union described themselves as “the catalyst for people who think traffic and the living environment should be more geared to the weakest groups in society.”^27^ The group wanted to stand up for what they called the “traffic poor,” stating that “it is no accident that the weakest in society are also ‘captive cyclists’ [*gedwongen fietsers*] and therefore also the weakest in traffic.”^28^ Schoolchildren, housewives, those who could not afford a car, or did not drive for other reasons, were all listed as groups that had to use the bicycle. At least some Dutch politicians agreed with this analysis. Social Democrat MP Jaap van der Doef saw the same “immobility” and “modern pauperization” caused by a society which one-sidedly provided access for car drivers, thus excluding all groups who could not (afford to) drive (Smaal 2012: 245). In Germany, the Allgemeine Deutsche Fahrrad-Club (ADFC, founded in 1979) similarly argued that cyclists were particularly vulnerable in traffic (Engler 2020: 359–60; Levels 2020).

Like Dooievaar and other bicycle advocates, the Cyclists’ Union used demonstrations to gain attention for its claims and put cycling back on the political agenda. In the early 1970s, activists in countries like the USA, UK, and France, organized bicycle processions to protest against the car (Furness 2010: 60–63). In the build-up to the official foundation of the Cyclists’ Union in October 1975, a series of mass demonstrations in big cities took place. Under the header “Amsterdam Autovrij” (*Amsterdam Car-Free*) three protest rides were first organized in 1974, drawing increasingly large crowds (Feddes & De Lange 2019: 78–80). Famously, in June 1977, during the “Wereldfietsdag” (*World Bicycle Day*), protesters lay down on the Museumplein portraying traffic victims. Over time this type of activism diminished somewhat, but still in 1982, the Amsterdam branch of the Cyclists’ Union organized a protest against the proposed closure of a cycling path on a bridge. Protest culture around the bicycle has declined ever since, in sharp contrast to other countries, such as the USA, where Critical Mass emerged in 1992 (Furness 2010: 78–107). This divergence can be attributed to the fact that cycling activists in countries like the Netherlands or Denmark, where cycling already had a place in mobility and politics, quickly gained attention for their problems. Protest, primarily meant to garner public support and pressure politicians, was no longer a necessary action strategy. In contrast, in more car-friendly countries with less of a cycling culture, protest remained often the only viable means of cycling activism (Furness 2010). In the Netherlands or Denmark, activists started to write alternative plans for cyclists that ended up forming input for policymaking. Martin Emanuel has shown how the Danish Cyclist Federation (DCF) developed a bicycle plan for Copenhagen. While not officially adopted, he writes that “planners did use the proposal as a blueprint to develop the bicycle network in Copenhagen in the following years.” (Emanuel 2019: 512). These different cycling activist cultures reflect both different cycling cultures and political traditions.

The main goal of Dutch cycling activists became the production of bottom up, user-generated knowledge to compensate for the knowledge gap that existed among urban planners regarding cycling. Once protest activities had succeeded in convincing government of the urgency of the problem, a dialogue between activists and government was established. The second step in the Cyclists’ Union action repertoire was to address the knowledge gap engineers had regarding cycling. Data about cyclists and their travel behavior and preferences was much scarcer than that about drivers. To the extent that they existed, government reports on cycling gave no insight into (subjective) dimensions of safety, comfort, route choice, and so on. The engineer P.B. van Gurp, director of the National Traffic Academy in Tilburg, drew attention to this in 1977 during a congress on cycling facilities. He noted that the interest in cycling was visible in the increasing stream of cycling memoranda (local) authorities published, but also concluded that: “A good picture of the most important cycling flows [*fietsstromen*] and bottlenecks is usually missing.” (Van Gurp 1978: 18). In addition, “there is insufficient understanding of the traffic safety problems which cyclists experience and undergo.” (Ibid.) It was exactly this knowledge gap which the Cyclists’ Union set out to address.

The Cyclists’ Union’s activism was based on the belief that the true experts on cycling were cyclists themselves. In cycling through urban streets every day, cyclists developed qualitative, experiential knowledge about the entire cycling experience, ranging from the safety of the route to the quality of the road surface and the parking facilities at public institutions. A true pro-cycling policy should be based on this type of knowledge, and not, or not exclusively, on quantitative traffic counts and studies of traffic flows. By virtue of its membership basis, consisting of ordinary cyclists, the Cyclists’ Union could provide this type of knowledge to local policymakers and secure its place in the policy coalition. In a guideline to the bottleneck approach, the Cyclists’ Union emphasized the danger of a quantitative approach. It was impossible to “objectively” state how much motorized traffic was acceptable on a road that cyclists also used. The dangerous appearance of objectivity that numbers provided could easily replace the lived experience of everyday cyclists, warned the Cyclists’ Union:

Numbers cannot lie, but liars can play with numbers! Do not let yourself be tempted into a quantitative approach in your confrontation with the government. It is of much greater importance to make clear that when it comes to cycling there is only one expert: i.e., *the cyclist himself/herself.* The cyclists themselves know best which situations are dangerous, which shorter connections are needed, which barriers need to be removed […] Where the government is doomed to fail given its technocratic approach, the Cyclists’ Union can be strong.^29^

It was therefore a central conviction of the Cyclists’ Union that making cycling policy without talking to cyclists themselves was ineffective and would not address the central concerns of cyclists. This was also why, for instance, some branches of the Cyclists’ Union queried their local councilmembers about their own preferred mode of transport. The Cyclists’ Union found it highly problematic if urban policymakers did not cycle because “only cyclists themselves know the problems of cycling through the city.”^30^

The process of acquiring legitimacy as a governance partner by gaining credibility as lay experts is, in an international perspective, not unique to the Dutch case (cf. Epstein 1995: 417–49). In his dissertation on the transnational circulation of French cycling activism, the French political scientist Maxime Huré discusses action groups’ self-legitimization in depth. He concludes that the role of user associations is important in the production of local expertise for municipal politics. (Huré 2013: 117) Huré’s focus is on the adaptation of expertise from countries with high cycling levels, like the Netherlands, to local circumstances in Belgium or France (Huré 2013: 158–59). What distinguishes the role of expertise as an action form in the Dutch context is that it is truly user-generated. This is due to the fact that the Dutch organizations operated in a context in which sizeable portions of the population still cycled. As a result, the type of knowledge these organizations created was not so much technical knowledge about how to construct cycling infrastructure or how to manage cycling governance. This knowledge was already largely available within Dutch government circles, where the construction of cycling infrastructure had continued without interruption from the 1920s, albeit with ebbs and flows. Instead, the Cyclists’ Union focused collecting and advocating for knowledge about the routes everyday cyclists took, the obstacles they faced on their journeys, and the bottlenecks that had to be prioritized as a result. In other words, the more advanced state of Dutch cycling was mirrored in the expertise they produced. Nevertheless, what unites the French action groups Huré studied with those across Europe is that the quest for legitimacy and recognition from policymakers in order to gain access to decision-making processes. In both French and Dutch cases, being seen as knowledgeable actors—acquiring “epistemic credentials”—is essential to their success (Stone 2012).

A few months before the founding of the Cyclists’ Union, in March 1975, activists already asserted that ideally local cyclists should come together to design the main cycling routes through their cities.^31^ In the second half of the 1970s, this is exactly what happened in many Dutch cities. The Cyclists’ Union branches often worked with what they called a “bottleneck memorandum” (*knelpuntennota*), quite literally mapping the greatest problems for cyclists in the city in detail. These were not so much concerned with congestion, as the word might suggest, but with any experience of cycling that was in some way compromised or constrained by road or traffic conditions which made cycling dangerous, unpleasant, or slower than it needed to be. The activists used this approach in many cities. One specific example is the town of Amersfoort, near Utrecht.^32^ The action began with the founding of a new group, which announced its presence by formulating a general critique of the city’s traffic policy in 1975. However, since this critique “was not very elaborate, we never heard anything.”^33^ Shortly thereafter, some council members demanded a cycling policy document from the city government which the local engineers duly produced. The Cyclists’ Union Amersfoort objected to this memorandum on the grounds that it was “seen and written from behind a car window.”^34^ In other words, and as discussed above, the perspective of cyclists on the traffic problems of the city were absent from this policy document. As it lacked input from cycling experience and did not account for data on cycling, the document did not satisfy cyclists’ concerns.

To formulate a better counterproposal, the activists handed out 3,000 questionnaires to cyclists in the city. The questionnaire was also printed in the free local paper and distributed at high schools.^35^ This resulted in 300 responses. The questionnaire asked cyclists to describe points where they felt unsafe, where road surfacing was poor, where waiting times at junctions and traffic lights were long, and so on. Meanwhile, the group also managed to convince the city council to postpone official discussion of the city’s memorandum so that the Cyclists’ Union could finish their proposal and deliver input for the final policymaking phase. Using the detailed input from the questionnaires, the activists used maps of the city to indicate which streets were unsafe and which crossings had to be redesigned in favor of cyclists, among other issues. They also devised an ideal cycling path network through the city. The report on the Amersfoort plan in the Cyclists’ Union internal magazine *De Ketting *(The Chain) included detailed instructions on how to compile, duplicate and distribute the memoranda, including the costs of this process.^36^ The Amersfoort branch stressed two points: First, good contacts with council members were indispensable. Without these connections, the activists would not have known what went on behind the scenes and they could not have delayed decision-making to allow for consideration of own memorandum. Secondly, they noted that their earlier attempt with a general critique of policy had been a failure, but the more concrete counterproposals of the second memorandum had been more effective.

The bottleneck memorandum was a popular means of addressing the problems of everyday cyclists. At the national meeting of the Cyclists’ Union on May 1, 1976, at least eleven local branches were in the process of compiling one: Amsterdam, Arnhem, Amersfoort, Delft, Enschede, Haarlem, ’s-Hertogenbosch, The Hague, Maastricht, Rotterdam and Utrecht.^37^ Within two years of its foundation, the Cyclists’ Union had widely adopted this effective method. As the Helmond branch noted in the mid-1970s: the questionnaires on bottlenecks that it collected were sent on directly to the municipality, “which usually then takes action.” They recommended this “simple but effective method” for Cyclists’ Union branches “which have good contact with the municipality.”^38^ To help those local branches, a guideline for constructing bottleneck memoranda was written around the same time. Apart from reflections on the past experiences of a number of branches, the guideline reflected more generally on the goal of this form of action. The text noted that the various branches of the Cyclists’ Union generated many proposals for cycling paths and other facilities but “lacked the data to elaborate these proposals, or provide proof for them.”^39^ The local Cyclists’ Union branch had to list all dangerous situations, barriers, missing links, and other deficiencies in the local cycling situation “in order to indicate where and how the government has to provide facilities for cyclists.”^40^ From the beginning, the Cyclists’ Union saw itself as fulfilling an important role in cycling governance, namely by providing local authorities with the knowledge needed to make better cycling policy.

That the Cyclists’ Union saw knowledge production as one of its key responsibilities can also be seen in their unsuccessful request to the national government for subsidy. This attempt, in 1979, though indicative of the Cyclists’ Union more conciliatory stance towards the government, was in no way unique: government subsidy for action groups was a common phenomenon in the Netherlands (Koopmans & Duyvendak 1992; Koopmans 1992). Nonetheless, the justification the group provided is illuminating. With only six (part-time) paid employees, the Cyclists’ Union considered its central office understaffed. This meant that “the ENFB [*Enige Echte Nederlandse Fietsersbond* = Cyclists’ Union] is not sufficiently able to fulfill a part of the tasks” they aspired to.^41^ The activities they listed were:

systematically processing relevant literature, orientation in activities and research of many organizations and institutions in the field of transport, translating for our purposes memoranda and research from among others the national government, reacting to national developments, setting up our own research, stimulating research in other organizations, attract interns and formulating study assignments.^42^

Developing all these knowledge-focused initiatives was necessary “for a mature functioning of our organization and for our role in the entire decision-making.”^43^ It is noteworthy that the Cyclists’ Union in this letter implies that power its ability to participate in the Dutch decision-making process was dependent on the knowledge they could bring to the table; thus the more playful happenings and protests of the early and mid-1970s were no longer held to be necessary or effective. In this development towards a more expertise-based type of activism, which consisted of producing reports and discussing them with governmental actors behind closed doors, the Cyclists’ Union quickly started to resemble the tourist organization ANWB in certain respects. Both organizations represented cyclists at different points in time, and both of them relied on expertise. Ebert (2010) has argued that in the Interbellum, the ANWB was much more powerful as a cycling advocate in the Netherlands than the bicycle clubs in Germany, which were divided along regional and class lines. Failing to form a unified front, these clubs could not claim to represent cyclists as a whole, something the ANWB could do and which helped it gain power. In the 1970s, if we extend her argument, one might expect that the existence of two, now rivalling organizations, might reduce the chances of success of the Cyclists’ Union. However, by 1970 the ANWB’s cycling advocacy had become a distant afterthought as the organization catered much more to drivers—the exact reason the Cyclists’ Union was founded.

In the final analysis, we need to distinguish between the everyday cyclists who may or (more likely) may not have been members of the Cyclists’ Union, and the leading figures within these action groups. The cyclists formed the true lay experts, while the activist leaders, who also cycled themselves and thus had this lay expertise as well, additionally possessed an intermediate level of expertise. None of them were trained traffic engineers, although some had training in architecture and urban planning (Dooievaar). Most leading figures, while academically trained, had backgrounds in the social sciences or humanities. This enabled them to write memoranda and policy documents in a style and jargon that might appeal to policymakers. It also meant that some of the activist leaders had a good personal relation, due to similar background and age, as some of the younger civil servants in local and national traffic departments. All this may explain why the Dutch activists were so successful, which is the topic of the final section of this paper.

## Success and State Relations

In a gradual process, urban policymakers worked with local activists to make cities safer and more attractive for cyclists since the 1970s. Coupled with traffic-calming measures and efforts to ban the car from city centres, this has achieved results. While in the early 1970s more than 500 cyclists died in traffic annually, this number started to decline to c. 400 around 1980 and 300 in 1990, before stabilizing around 200 since the 2000s.^44^ Investments in cycling paths, tunnels, bridges, and parking places helped separate bicycle from car traffic, shortened cycling travel distances, and supported the bicycle-train combination as an alternative to driving (Dekker 2021: 267–305). From a governance standpoint, this result is attributable to a close relationship between activists, politicians, and state engineers.

From their side, the activists by and large were willing to work with the government. Apart from their empirical input, they were also willing to be pragmatic and moderate in rhetoric. Early activists, like André Guit within the Cyclists’ Union Amsterdam quickly started to think strategically (see also Feddes & De Lange 2019). In 1978, he was involved in the construction of memoranda listing all major and minor bottlenecks for cycling in the city—a form of activism more geared towards cooperation than disruption. The social-democrat alderman Michael van der Vlis supported cycling and his party had prepared a similar memorandum, so there was a clear synergy between the activists and the policymakers.^45^ However, the activists themselves were divided over this: when they were invited to participate in the municipal Working Group Cycling, some members supported this while others wanted to remain a more activist, outsider group, leading to “principled and fierce” debate within the Amsterdam Cyclists’ Union.^46^ Looking back on these years, Guit has remarked that while they were “very much anti-car”, for strategic reasons they “prudently did not spread this message”.^47^ Dooievaar activist Hans van Beek also was opposed to radical and swift change, believing that “through education and discussion the mentality has to be adjusted” before implementing sweeping measures.^48^ Being pragmatic in order to achieve as much as possible was how many of the activists operated in Dutch cities.

Because activists offered this knowledge to state officials broadly sympathetic to cycling, this work can be seen as indirectly responsible for the redistribution of urban space in favour of cyclists since the 1970s. In the 1970s activists and citizens explicitly politicized many issues which up to that point had been governed in a technocratic way (Aerts et al. 1999: 297–306). Historian James Kennedy in particular has argued that Dutch politicians and officials were so taken aback by this wave of citizen activism that they gave in or even proactively implemented citizen demands (Kennedy 1995; Kaal 2011). The mid-1970s, when the Netherlands had a progressive social-democratic government led by Joop Den Uyl, was a period full of opportunity for action groups. State officials and politicians were particularly open to ideas of participation in this decade (Kickert 2003: 122). During the annual meeting of the Cyclists’ Union in 1977, for example, a debate was organized by a municipal alderman, while the panel included highly ranked officials and one member of parliament.^49^ A significant number of Dutch engineers and policymakers were willing to take cycling seriously and acknowledged the activists’ valuable contribution to policymaking. The cultural frame of cycling as typically Dutch undoubtedly also played a role in this receptiveness (Ebert 2010; Oosterhuis & Stoffers 2009; Oosterhuis 2016).

Scholars have noted that in a comparative European perspective, the Dutch political system has certain characteristics and informal norms that make it relatively easy for social movements to exert some influence. These include that fact that civil servants remain in their positions when government changes, creating stability and a high level of professionalism. Activists could and did gain occasionally gain employment within this system. In addition, the low electoral threshold for new parties meant politics had to be more responsive to public demands. New activist movements could form their own political parties and gain access to Parliament relatively easily. To pre-empt this, established parties often adopted the program of activists in order to obtain votes. Additionally, even the more rigid parts of the political system are softened by an informal culture of consensus and compromise (Duyvendak & Koopmans 1992). Common strategies within the Dutch political system were facilitation (by subsidizing action groups), co-optation (giving them a place in advisory bodies) and assimilation (arriving at shared problem definitions) (Van der Heijden 1992: 81). In places where policymakers were open to activists, such groups could quickly professionalize, as Astrid Kirchhof has also shown for environmental action groups in West Berlin (Kirchhof 2015).

Dutch politicians and engineers took citizens’ ideas about their living environment seriously. They appreciated the specific input and complaints of citizens. G.H.A. Hoogenboom (1972), an engineer working for Amsterdam’s Traffic Office (*Verkeersbureau*), stressed the need for specificity again in 1972, saying: “It is important for action groups not to aim their actions at city-wide issues, but that they aim at issues that they have to deal with in their street.”^50^ In an article in *De Tijd *(the newspaper promoting “Stop de Kindermoord”), he advised the activists to develop expertise and do as much groundwork for the construction of cycling paths as possible: time-consuming expropriation procedures could be prepared by activists talking to land owners in advance, activists could also design a number of alternative routes for cycling paths. “If you take up a part of the preparatory work and remove obstacles, you can accelerate the preparation.”^51^ Jan de Ruiter, a council member for a confessional party in Heemstede municipality, noted in 1972 that it was “no surprise that it is precisely on traffic that such an interesting dialogue with sections of the population has been established.” Because it affected everyone directly, traffic was an issue that generated a lot of interest among citizens. For this council member it was “of paramount importance” that representatives listen to local action groups like The Troublesome Cyclist (*De Lastige Fietser*).^52^

While a majority of Cyclists’ Union figures chose cooperation over confrontation, there also was dissent. The Maastricht branch argued that “we are being completely taken in by the cycling path plans in many cities.”^53^ In their view, a pro-cycling policy had to pursue the goal of lowering levels of car ownership and deploy confrontational strategies that the Cyclists’ Union—and Dutch mobility policy—typically avoided. Similarly, the Nijmegen branch argued in 1980 that it eschewed participation in municipal advisory groups so that it could still organize street protests. Participating in local political was time-consuming and encouraged the view that “the municipality doesn’t expect tough action because you sit on the committee.”^54^ This debate occurred in many traffic action groups outside the Netherlands as well. In the US, the group Transportation Alternatives (TA) was divided between “those who wanted to continue with anti-automobile advocacy and those who wanted TA to pursue a more positive direction that emphasized the benefits of cycling and a less contestatory anti-car rhetoric.” (Furness 2010: 63).

A high degree of mobility between activism and government also paved the way for activists’ success (see also Feddes & De Lange 2019). Although most activists were young, they shared a similar socio-economic background with their interlocutors within the professional bureaucracy. Cities like Delft hired young traffic engineers with new ideas about urban planning [*woonerf*], traffic calming, and cycling facilities. Eisse Kalk, the inspirator of Dooievaar, took a job with the Amsterdam city government in 1981 to reform policymaking from the inside. By working from inside the government bureaucratic apparatus, Kalk hoped to achieve more.^55^ Steven Schepel, who was a prominent member of Stop de Kindermoord in the 1970s, started to work as a government official in traffic safety in the 1980s, first in Rotterdam, then later in Amsterdam. He ended his career as an official in the Ministry of Public Works, where he worked on traffic safety programs. Again, the Danish case proves remarkably similar. Martin Emanuel argues that the Copenhagen city government lacked planning expertise and therefore looked to “former bicycle activists” who took a job with the city’s planning office (Emanuel 2019: 515). These individuals brought their cycling expertise to these governmental institutions and helped to create a more cycling-friendly planning culture within traffic departments.

For Dutch activists, gaining initial entrance into local politics was relatively easy. However, activists found that translating these contacts into concrete improvements in the streets could take frustratingly long. The slow planning procedure of civil servants clashed with the urgency of cyclists’ demands. Politicians spoke out in favor of cycling*, *but that was not enough. In 1980, at a Cyclists’ Union event, the pro-cycling The Hague council member R.W. Heus (a conservative liberal and engineer by training) and his social democrat colleague Burgers-Molendijk acknowledged this gap between political ambitions and implementation: “The plans that civil servants at city hall design do not connect enough to the ideas in the city council.” The true “promotion of slow traffic” by public works engineers did not conform with the wishes of legislators and activists.^56^ By 1982, an Amsterdam cycling action group supported by the Cyclists’ Union organized its first mass demonstration in four years because of this inertia. Dealing with experienced bureaucrats presented something of a learning curve for young activists who had to learn not to “be brushed off with pseudo commitments.”^57^ The Cyclists’ Union deliberately chose to cooperate with city government because it wanted “to get out of the phase of hollow phrases” to achieve something.^58^ Though they would eventually secure results, making Dutch cities attractive for cyclists was a process that took decades, rather than a few years.

Besides the slow nature of change, some activists for the democratization of traffic also criticized the superficial nature of participation. Eisse Kalk in particular pointed out that while participation was the watchword of the day, the government held a rather narrow conception of the term. Kalk distinguished between “participation-for-the-government” and “participation-for-residents.” (Kalk 1978). Kalk argued the Dutch government used the former, seeing participation as a “social technique” to gain insight into public opinion and to more effectively govern, rather than viewing it as a tool to learn about the genuine needs and desires of citizens, and in particular “the weakest groups of society.” By making policy based on the data collected in the Cyclists’ Union surveys, Dutch traffic planners gained valuable insight and data without requiring own civil servants to gather this data or hire expensive consultants. Did this represent merely tweaking a system in which the basic tenets remained unchanged? The introduction of national subsidies for urban cycling in 1975 represented a significant victory for decentralized mobility policy and jumpstarted the construction of urban cycling infrastructure (Dekker 2021). More detailed research within particular cities could demonstrate the extent to which citizen complaints were indeed taken seriously.

While historical analyses of cycling activism in other European countries is scarce, we can still argue that some of the conditions for the success of Dutch cycling activism were lacking elsewhere. Ebert’s argument that German cycling clubs in the interwar period lacked political clout due to their failure to present a unified front for cycling may also apply to the more recent cycling activism of the German ADFC. Further research is needed to substantiate this. However, the ADFC and cycling advocates elsewhere, with the exception of Denmark, do not represent as large a share of the population as Dutch cycling activists. This question of scale already presents a significant obstacle to political power. It also made it more difficult to “source” experiential data from everyday cyclists and translate this into demands for cycling policies, as this article has demonstrated happened in the Netherlands. Additionally, it is also clear that cycling in most other European countries did not carry the cultural significance it did in the Netherlands (Ebert 2010; Kuipers 2013), making policymakers less receptive to cyclists’ demands.

All in all, we can conclude that in the 1970s a consensus emerged in the Netherlands that cycling would not disappear as a mode of transportation; instead it came to be seen as valuable and meriting promotion. In order to achieve this, engineers, politicians and activists agreed that more and better cycling infrastructure and parking facilities were needed. This article has shown how the political process of making claims for cycling and convincing policymakers and engineers of the importance revolved around the claim that ordinary cyclists were experts. They knew best what made everyday bicycle commuting safer and ultimately a more viable alternative to cars and public transit. The Cyclists’ Union, in its role as an intermediary between users and government, collected this counter-expertise and, working with state officials, set in motion a process in which Dutch cities have created some of the most bicycle-friendly traffic environments in the world.

## Footnotes

1 For a fuller discussion of Ebert and the historiography of Dutch cycling in general, see Dekker (2021), Introduction and Part One.

2 Data from Stichting Wetenschappelijk Onderzoek Verkeersveiligheid SWOV. See: https://www.swov.nl/feitenencijfers/verkeersveiligheidscijfers-verkeersongevallen > Historische Reeksen > Doden naar leeftijd en vervoerswijze vanaf 1950 [last accessed 06-01-2021]

3 Joop Daalmeijer, “We moeten bewuster leven ook met het verkeer,” *Het Nieuwe Stadsblad*, 19-05-1965 (1965). Jaspers placed a piece of cardboard that he was carrying around under the victim’s head. This bloodied cardboard he then wanted to exhibit as a “national monument for traffic victims.”

4 “Auto kapot rameien voor ‘veilig verkeer’,” *Rotterdamsch Parool/De Schiedammer*, 22-05-1965 (1965); “Happening voor veilig verkeer Schiedam,” *Het Vrije Volk*, 22-05-1965 (1965); “Hysterische ‘kunstrakkers’ slopen Nieuwlandse galerie,” *Het Nieuwe Stadsblad*, 25-05-1965 (1965). Jaspers’ interest in traffic safety seems to have been relatively short-lived; he would later move to Groningen where he became active in the squatters’ movement as well as the peace movement.

5 “Zieke kabouter weggebracht: In drie steden acties,” *De Volkskrant*, 13-04-1970, (1970: 3).

6 Cf. Geert Mak (1992), “Anatomie van een romantische stadsrebellie,” *NRC Handelsblad*, 03-10-1992.

7 In one symbolic procession, the group hired a stuffed stork and carried it around as if to bury it (Interview Hans van Beek and Leo Hamer, The Hague, 05-02-2019). See on the cycling history of The Hague Eric Berkers, Frans Botma, and Ruth Oldenziel, *Cycling Cities: The Hague Experience* (Eindhoven: Foundation for the History of Technology, 2018).

8 “Burger wordt dupe van groeibeleid,” *Trouw*, 27-11-1972 (1972), T12; N.H.J. de Bruijn, “Wie mee wil praten moet het nu vertellen,” *NRC Handelsblad*, 05-02-1973 (1973: 8).

9 Paul Kouwenberg, “Inspraak-begeleider Eisse Kalk: Democratie verbeteren kost tijd,” *Volkskrant*, 08-03-1975 (1975: 19).

10 Werkgroep Dooievaar, “Weg fiets? Nee fietswegen!,” *Tijdschrift voor Verkeerstechniek* 24, no. 11 (1973), 552–53.

11 GADH (Municipal Archives The Hague), Archive Dooievaar (0772-01), inv. No. 3, letter E. Kalk to F.J. Smits, 20-07-1972. In this letter Kalk (of the action group Werkgroep 2000) explained to Smits, the director of the Rotterdam Architecture School, why the somewhat unorthodox project of the students had relevance for the profession. Kalk and Werkgroep 2000 were involved in experiments with increasing participation in local policymaking and Dooievaar formed an early partnership with them.

12 Werkgroep 2000 in association with Werkgroep Dooievaar, “Fietser … kop op!,” *Katernen 2000 1974*, no. 9–10 (1974), 6.

13 Interview Hans van Beek and Leo Hamer, Dooievaar founders, The Hague, 05-02-2019.

14 GADH, Archive Dooievaar (0772-01), inv. no. 5, “Samenvatting studie 1970–1972”, p. 20.

15 GADH, Archive Dooievaar (0772-01), inv. no. 5, letter Dooievaar to The Hague city council, 01-11-1972.

16 Dooievaar, “Weg fiets? Nee fietswegen!.”

17 “Dooievaar wil Hagenaar weer laten fietsen,” *De Volkskrant*, 24-02-1973, (1973: 39).

18 “Protest tegen slechte klimaat voor fietsers,” *NRC Handelsblad*, 12-06-1973, 10.

19 Ibid.

20 GADH, Archive Dooievaar (0772-01), inv. no. 4 contains many articles on Dooievaar by the journalist Jek Kleijn. Similarly, journalist Lisette Lewin gave the Amsterdam action group De Lastige Amsterdammer multiple long interviews in Algemeen Handelsblad and later NRC Handelsblad: Lewin, “Lastige Amsterdammer Henk Bakker: ”Alles is te regelen als eerst die auto maar weg is”.” (1970); Lisette Lewin, “Fielt,” *NRC Handelsblad*, 11-11-1972 (1972a); Lisette Lewin, “Henk Bakker: metro is Lammers’ struikelblok,” *NRC Handelsblad*, 20-11-1972 (1972b); Lisette Lewin, “Amsterdammer is weer lastig: metro geen comfort,” *NRC Handelsblad*, 26-01-1973 (1973).

21 Ibid.

22 In 1975, Stop de Kindermoord also published a brochure which documented tips and best practices for activists: Stop de Kindermoord, *Handleiding voor Inspraak en Aktie* (Amsterdam: Pressiegroep Stop de Kindermoord 1975).

23 Werkgroep 2000 in association with Werkgroep Dooievaar, “Fietser … kop op!.” A digitalized version can be accessed at https://fietsstadamsterdam.nl/wp-content/uploads/2019/05/1974FI1.pdf. Dooievaar also made a video demonstrating their process. It can be accessed through https://haagsgemeentearchief.nl/mediabank/beeldcollectie by searching for “Karre maar.”

24 In this plan, the emphasis was on cycling paths. As architects the The Hague activists were also very interested in cycling parking, both at home and at public venues (Interview Hans van Beek and Leo Hamer, The Hague, 05-02-2019; “Het probleem van de fiets: het opbergen,” *De Tijd*, 24-02-1973, 1973: 3.).

25 I am using the term Cyclists’ Union throughout, but the organization underwent several name changes. Initially founded as ENWB (an acronym of Eerste Enige Echte Nederlandse Wielrijders Bond [First Real Only Dutch Cyclists’ Union]), it changed its name in 1979 into ENFB in reaction to pressure from the rivalling ANWB (see below). The name had consciously been adopted as an accusation towards the ANWB for neglecting its original status as a bicycle club

26 NA (National Archives), Archive Stop de Kindermoord (2.19.198), inv. no. 241, letter F.H. Markerink to ENWB, 08-10-1975.

27 IISG (International Institute for Social History), Archive Cyclists’ Union (ARCH01969), inv. no. 1, “Notitie de E.N.W.B. en de bierkaai”, 05-04-1978.

28 Ibid.

29 IISG, Archive Cyclists’ Union, inv. no. 1, Christiaan la Poutré, “Inventarisatie knelpunten”, p. 2. Underlined in original.

30 Archief Eemland, Archive ENWB Amersfoort (242), inv. no. 3, letter Cyclists’ Union to Mayor and Alderman, 18-01-1976.

31 Elly van der Ster, “Hoe sterk is de eenzame fietser,” *De Volkskrant*, 29-03-1975, 35.

32 “Afdeling Amersfoort: ‘Bericht over het ontstaan van een fietsrapport’,” *De Ketting* 1 (1976), 2–3.

33 Ibid.

34 Ibid.

35 IISG, Archive Cyclists’ Union, inv. no. 1, Christiaan la Poutré, “Inventarisatie knelpunten”, 5.

36 “Afdeling Amersfoort: ‘Bericht over het ontstaan van een fietsrapport’.”

37 IISG, Archive Cyclists’ Union, inv. no. 1, “Verslag ledenraad dd. 1 mei 1976 E.N.W.B. te Utrecht”. The Hague’s plan was based on 175 cyclists’ experiences (GADH, Archive Dooievaar (0772-01), inv. no. 10). The Amsterdam branch devised a memorandum in 1977. This document positioned itself against the Traffic Circulation Plan of the city, which proposed a city-wide network of separated cycling path. The Amsterdam Cyclists’ Union argued that not only was this unrealistic, but also unnecessary, since many bottlenecks for cyclists could be solved with relatively simple and cheap means. In addition, they argued that cycling should be safe everywhere and should not be dependent on the existence of separated cycling paths. The whole infrastructure of the city had to be rethought from the perspective of cyclists and not automobile driver’s (Feddes and De Lange, *Fietsstad Amsterdam*, 91–94).

38 IISG, Archive Cyclists’ Union, inv. no. 1, Christiaan la Poutré, “Inventarisatie knelpunten”, 6.

39 IISG, Archive Cyclists’ Union, inv. no. 1, Christiaan la Poutré, “Inventarisatie knelpunten”, 1.

40 IISG, Archive Cyclists’ Union, inv. no. 1, Christiaan la Poutré, “Inventarisatie knelpunten”, 1.

41 NA, Archive Second Chamber of Parliament (2.02.28), inv. no. 6272, letter ENWB to Minister of Public Works, 18-05-1979.

42 NA, Archive Second Chamber of Parliament, inv. no. 6272, letter ENWB to Minister of Public Works, 18-05-1979.

43 NA, Archive Second Chamber of Parliament, inv. no. 6272, letter ENWB to Minister of Public Works, 18-05-1979.

44 Data from Dutch Road Safety Institute SWOV (Stichting Wetenschappelijk Onderzoek Verkeersveiligheid). See: https://www.swov.nl/feitenencijfers/verkeersveiligheidscijfers-verkeersongevallen and https://theseus.swov.nl/single/?appid=5dbac35a-5fbd-401f-b711-682176941688&sheet=jvpBF&opt=currsel%2Cctxmenu for the data from 1996 and later.

45 “Meter voor meter fietspaden bevechten,” *Op eigen kracht: Blad van de afdeling Amsterdam van de Fietsersbond*, no. 77 (2009), 12.

46 Ibid.

47 Ibid., 13.

48 “Dooievaar wil Hagenaar weer laten fietsen,” (1973: 39).

49 Metselaar (1977), “De vuist van de gemangelde fietsers,” 5.

50 “Zo wil Amsterdam zijn verkeersknooppunten doorhakken,” *Het Parool*, 23-02-1974, (1974: 3).

51 G.H.A. Hoogenboom, “Actie is zinvol maar vereist veel geduld,” *De Tijd*, 24-10-1972 (1972: 3).

52 Krantenviewer Noord-Hollands Archief, Raadsnotulen Heemstede, 14-12-1972, p. 390. https://nha.courant.nu/issue/RNH/1972-12-14/edition/Notulen/page/19?query=%22lastige%20fietser%22&sort=relevance

53 “ENWB afd. Maastricht,” De Ketting 1 (1976): 5. Eventually some members who preferred action became disillusioned and left the organization: Gert van Engelen, “Overleggen is niet fout, ingepakt worden wel,” De Vogelvrije Fietser 16, no. 1 (1990): 24–25.

54 Paulien Osse and Theo de Jong, “‘Als je in de commissie zit, verwacht de gemeente dat je geen harde acties meer voert’,” Vogelvrije Fietser 5, no. 4 (1980): 18–19, here 19.

55 “‘Burger moet echt kunnen meespreken’,” *Het Parool*, 24-03-1981 (1981), 7; “Eisse Kalk: ‘In dit werk is een brede rug nodig’,” *Het Parool*, 13-04-1983, (1983: 6).

56 “Fietsers hebben weinig aan de politiek,” Trouw, June 10, 1980.

57 Paul Godefrooij and Toni Niël, “‘Rijkswegen, dat is de grootste prioriteit in Brabant’,” De Vogelvrije Fietser 9, no. 3 (1984): 18–19, here 19.

58 “Fietsvriendelijke stad is nog toekomstmuziek,” Trouw, May 14, 1982; Jan Ploeger, “De komende tien jaar zouden wel eens oogstjaren kunnen worden,” De Vogelvrije Fietser 11, no. 1 (1985): 6–7.
